# Phage Therapy Is Effective against Infection by *Mycobacterium ulcerans* in a Murine Footpad Model

**DOI:** 10.1371/journal.pntd.0002183

**Published:** 2013-04-25

**Authors:** Gabriela Trigo, Teresa G. Martins, Alexandra G. Fraga, Adhemar Longatto-Filho, António G. Castro, Joana Azeredo, Jorge Pedrosa

**Affiliations:** 1 Life and Health Sciences Research Institute (ICVS), School of Health Sciences, University of Minho, Braga, Portugal; 2 ICVS/3B's - PT Government Associate Laboratory, Braga/Guimarães, Portugal; 3 Institute for Biotechnology and Bioengineering (IBB), Centre of Biological Engineering, University of Minho, Campus de Gualtar, Braga, Portugal; 4 Laboratory of Medical Investigation (LIM), Faculty of Medicine, University of São Paulo, São Paulo, Brazil; 5 Molecular Oncology Research Center, Barretos, São Paulo, Brazil; Fondation raoul Follereau, France

## Abstract

**Background:**

Buruli Ulcer (BU) is a neglected, necrotizing skin disease caused by *Mycobacterium ulcerans*. Currently, there is no vaccine against *M. ulcerans* infection. Although the World Health Organization recommends a combination of rifampicin and streptomycin for the treatment of BU, clinical management of advanced stages is still based on the surgical resection of infected skin. The use of bacteriophages for the control of bacterial infections has been considered as an alternative or to be used in association with antibiotherapy. Additionally, the mycobacteriophage D29 has previously been shown to display lytic activity against *M. ulcerans* isolates.

**Methodology/Principal findings:**

We used the mouse footpad model of *M. ulcerans* infection to evaluate the therapeutic efficacy of treatment with mycobacteriophage D29. Analyses of macroscopic lesions, bacterial burdens, histology and cytokine production were performed in both *M. ulcerans*-infected footpads and draining lymph nodes (DLN). We have demonstrated that a single subcutaneous injection of the mycobacteriophage D29, administered 33 days after bacterial challenge, was sufficient to decrease pathology and to prevent ulceration. This protection resulted in a significant reduction of *M. ulcerans* numbers accompanied by an increase of cytokine levels (including IFN-γ), both in footpads and DLN. Additionally, mycobacteriophage D29 treatment induced a cellular infiltrate of a lymphocytic/macrophagic profile.

**Conclusions/Significance:**

Our observations demonstrate the potential of phage therapy against *M. ulcerans* infection, paving the way for future studies aiming at the development of novel phage-related therapeutic approaches against BU.

## Author Summary

Buruli Ulcer (BU), caused by *Mycobacterium ulcerans*, is a necrotizing disease of the skin, subcutaneous tissue and bone. Standard treatment of BU patients consists of a combination of the antibiotics rifampicin and streptomycin for 8 weeks. However, in advanced stages of the disease, surgical resection of the destroyed skin is still required. The use of bacterial viruses (bacteriophages) for the control of bacterial infections has been considered as an alternative or a supplement to antibiotic chemotherapy. By using a mouse model of *M. ulcerans* footpad infection, we show that mice treated with a single subcutaneous injection of the mycobacteriophage D29 present decreased footpad pathology associated with a reduction of the bacterial burden. In addition, D29 treatment induced increased levels of IFN-γ and TNF in *M. ulcerans*-infected footpads, correlating with a predominance of a mononuclear infiltrate. These findings suggest the potential use of phage therapy in BU, as a novel therapeutic approach against this disease, particularly in advanced stages where bacteria are found primarily in an extracellular location in the subcutaneous tissue, and thus immediately accessible by lytic phages.

## Introduction

Buruli Ulcer (BU), caused by *Mycobacterium ulcerans,* is an emerging, devastating skin disease reported in more than 30 countries, mainly in West Africa [1], [2]. BU is characterized by different clinical forms, including nonulcerative subcutaneous nodules, papules, edema, and plaques that can progress to necrotic ulcerative forms. The pathogenesis of BU is associated with mycolactone, a lipidic exotoxin presenting cytotoxic and immunosuppressive properties [3]–[7]. Prevention is difficult as little is known about disease transmission, although it has been shown that *M. ulcerans* is an environmental pathogen [8]–[10], and no vaccine is available.

Since 2004, the World Health Organization (WHO) recommends the treatment of BU with a combination of rifampicin and streptomycin (RS) [11]. Nevertheless, this treatment presents several limitations: (i) it does not resolve extensive lesions (as a result, surgery is the only alternative) [12]; (ii) the long period of administration of streptomycin by muscular injection demands skilled personnel; (iii) it is associated with adverse side effects [13], [14] leading to poor compliance; and (iv) importantly, it may lead to the occurrence of paradoxical reactions associated with the worsening of the lesion and/or the appearance of new lesions [14]–[18].

Bacteriophages (phages) have been proposed to treat human bacterial infections since their discovery in the early 20^th^ century [19]. Several well controlled studies in both animal models and human infections have successfully applied phage therapy to several types of bacterial infections, demonstrating its potential as an antibacterial therapy *in vivo* [20]–[30] Additionally, in the UK, the first phase II clinical trial performed under European regulations on phage treatment of chronic otitis has open the door for novel phage-based human applications [31].

Phage therapy presents several potential advantages for the treatment of BU patients, namely phages present lytic activity against extracellular bacteria which predominate in advanced lesions; phages may be used for the treatment of ulcerative lesions where the necrotic infection site would be accessible; and phages may be administered topically [28].

In the present study, following the screening of the lytic activity of several mycobacteriophages, the therapeutic effect of the selected mycobacteriophage D29 was evaluated against *M. ulcerans* in the mouse footpad model of infection. The progression of macroscopic/microscopic pathology and bacterial load, as well as the cytokine profile, in both the footpad and the draining lymph node (DLN), were evaluated after mycobacteriophage D29 administration.

## Materials and Methods

### *In vitro* mycobacteriophage activity against *M. ulcerans*

Mycobacteriophages, kindly provided by Dr. Graham F. Hatfull from the Pittsburgh Bacteriophage Institute and Department of Biological Sciences, University of Pitsburgh, were screened against *M. ulcerans* isolates. In order to select mycobacteriophages active against *M. ulcerans* strains, we first selected representative isolates of *M. ulcerans* from endemic BU areas, based on their genetic and phenotypic characteristics, including the type of mycolactone produced [3], [32], [33] and their virulence in mice [7], [34] (see Table 1). The strains were obtained from the collection of the Institute of Tropical Medicine (ITM), Antwerp, Belgium.

**Table 1:** Characteristics of *M. ulcerans* isolates used.

*M. ulcerans*	Origin	Geographical origin	Type of Mycolactone
98-912	Ulcer	China	D
97-1116	Plaque	Benin	A/B
1615	Ulcer	Malaysia	A/B
94-1331	nd	Papua New Guinea	A/B
94-1327	Ulcer	Australia	C
5114	Ulcer	Mexico	-
00-1441	Ulcer	Benin	A/B
94-1324	Aquatic insect	Australia	C
03-216	Ulcer	Benin	A/B

nd, not determined; -, Mycolactone negative.

This host-range determination was done by adapting a spot-test technique described elsewhere [35], [36]. Briefly, *M. ulcerans* was grown to an OD_600_ of 1.0 and clumps were dispersed by passing the bacterial suspension several times through a 25-gauge needle. The suspension was plated on Middlebrook 7H9 agar medium (Becton, Dickinson and Company). For each mycobacteriophage, serial dilutions were prepared in phage buffer (MPB) (10 mM Tris, pH 7.5, 1 mM MgSO_4_, 70 mM NaCl) and were plated onto the *M. ulcerans* lawn and the spots were allowed to dry completely. Plates were incubated at 32°C for approximately 6–8 weeks.

### Animals

A total of 120 (*per* experience) eight-week-old female BALB/c mice were obtained from Charles River (Barcelona, Spain) and were housed under specific-pathogen-free conditions with food and water *ad libitum*.

### Footpad mouse model of *M. ulcerans* infection

*M. ulcerans* 1615 is a mycolactone A/B producing strain isolated in Malaysia from an ulcerative case [7]. The isolate was grown on Middlebrook 7H9 agar medium at 32°C for approximately 6–8 weeks. For the preparation of inoculum, *M. ulcerans* was recovered, diluted in phosphate-buffered saline (PBS) and vortexed using glass beads. The number of acid-fast bacilli (AFB) in inocula were determined as described previously using Ziehl-Neelsen (ZN) staining [37]. Mice were infected in the left hind footpad with 0.03 ml of *M. ulcerans* suspension containing 5.5 log_10_ AFB.

### Treatment of *M. ulcerans*-infected mice with mycobacteriophage D29

D29 particles were propagated in *Mycobacterium smegmatis* mc^2^155 (ATCC), as described elsewhere [36]. In brief, approximately 10^5^ phage particles and 250 µl of *M. smegmatis* mc^2^155 (ATCC) (OD_600_ of 1.0) were plated on Middlebrook 7H9 overlays (0.6% agar) and incubated at 37°C overnight. Phage particles were extracted with 3 ml of MPB and harvested filtering through a 0.2 µm pore-size filter. Phages were concentrated through polyethylene glycol (PEG) precipitation and purified using a CsCl equilibrium density gradient centrifugation. Phage titers (PFU/ml) were determined by serial dilution and plaque assays by the soft overlay technique with some modifications [35]. Briefly, phage dilutions were spotted onto Middlebrook 7H9 overlays (0.6% agar) with *M. smegmatis* mc^2^155 (ATCC) and incubated at 37°C overnight.

The treatment was initiated at day 33 post-infection, when the footpad of mice were swollen to 3.0 mm, and was performed by subcutaneous injection in the infected footpad with a single dose of mycobacteriophage D29 containing 8 log_10_ PFU. MPB was given to control (non-treated) mice.

### Assessment of footpad swelling

Footpad swelling was monitored throughout the experiment, as an index of lesion development, by using a caliper to measure the diameter of the frontal area of the footpad. For ethical reasons, the non-treated mice were sacrificed after the emergence of ulceration at day 68 post-infection, and no further parameters were evaluated for this group.

### Bacterial and phage growth

*M. ulcerans* growth and phage proliferation were evaluated in footpad tissues and in the DLN. Briefly, footpad tissue specimens were minced, resuspended in PBS (Sigma) and vortexed with glass beads to obtain homogenized suspensions. DLN were homogenized, the cell numbers were counted and then suspensions were lysed with saponin 0.1%. Serial dilutions of the footpad and DLN homogenates were plated on Middlebrook 7H9 agar medium. *M. ulcerans* numbers were counted after 6 to 8 weeks of incubation at 32°C and expressed as colony forming units (CFU/ml). Homogenized samples were also centrifuged for 10 min at 5000 rpm, supernatant was used for phage determination by the soft overlay technique [35] and expressed as plaque forming units (PFU/ml). Phage dissemination was also investigated by detecting phages in the spleen and sera of mice.

### Detection of cytokines

The levels of the cytokines tumor necrosis factor (TNF), interleukin (IL)-6, gamma interferon (IFN-γ) and IL-10 in the supernatant of homogenized suspensions from DLN and footpad tissues of control-infected and mycobacteriophage D29 treated mice were quantified by using a Quantikine Murine ELISA kit (eBioscience Inc), according to the manufacturer's instructions.

### Histological studies

Mouse footpads and DLN were harvested, fixed in 10% phosphate-buffered formalin and embedded in paraffin. Light microscopy studies were performed on tissue sections stained with hematoxylin and eosin (HE) or Ziehl-Neelsen (ZN). Images were obtained with an Olympus BX61 microscope.

### Statistical analysis

Differences between the means of experimental groups were analyzed with the two-tailed Student t test. Differences with a P value of ≤0.05 were considered significant.

### Ethics statement

This study was approved by the Portuguese national authority for animal experimentation Direção Geral de Veterinária (ID: DGV 594 from 1st June 2010). Animals were kept and handled in accordance with the guidelines for the care and handling of laboratory animals in the Directive 2010/63/EU of the European Parliament and of the Council.

## Results

### Mycobacteriophage D29 shows a broad lytic activity against *M. ulcerans* isolates *in vitro*

We first tested the lytic activity of different mycobacteriophages against several *M. ulcerans* isolates. The results for the plaque formation on the tested *M. ulcerans* strains are given in Table 2. We observed that some phages were more strain-specific, such as the phages Adjutor, Kostya and Brujita, and others presented a more narrow lytic host range spectrum (L5, Chah and Phaedrus). A cluster of three phages, namely D29, Bxz2 and Tweety, displayed the broadest lytic host range spectrum and highest lytic activity against representative strains of *M. ulcerans*. In line with a previous report [36], D29 phage showed the broadest lytic host range spectrum amongst the tested mycobacteriophages, affecting *M. ulcerans* isolates with genetic heterogeneity, variable phenotypic characteristics and from different geographic origins (Table 1). Based on these results, we selected mycobacteriophage D29 for *in vivo* therapeutic studies against infection with *M. ulcerans* 1615, a well characterized and stable strain that presents a mycolactone profile identical to that of African strains [32].

**Table 2:** Sensitivity of phages to *M. ulcerans* isolates.

	Phage								
*M. ulcerans*	D29	Bxz2	L5	Tweety	Chah	Adjutor	Kostya	Phaedrus	Brujita
98-912	P	-	-	-	P	-	P	-	-
97-1116	P	P	P	-	-	-	-	-	-
1615	P	-	-	-	-	-	-	-	-
94-1331	P	-	-	-	-	-	-	-	-
94-1327	-	-	-	P	-	P	-	-	-
5114	P	P	-	P	-	-	-	P	-
00-1441	P	P	-	P	P	-	-	-	P
94-1324	P	P	P	P	-	-	-	P	-
03-216	P	-	-	-	-	-	-	-	-

P-plaque formation.

### Treatment with mycobacteriophage D29 prevents ulceration caused by *M. ulcerans* and decreases the bacterial load in both the footpad and the DLN

**Figure 1:**
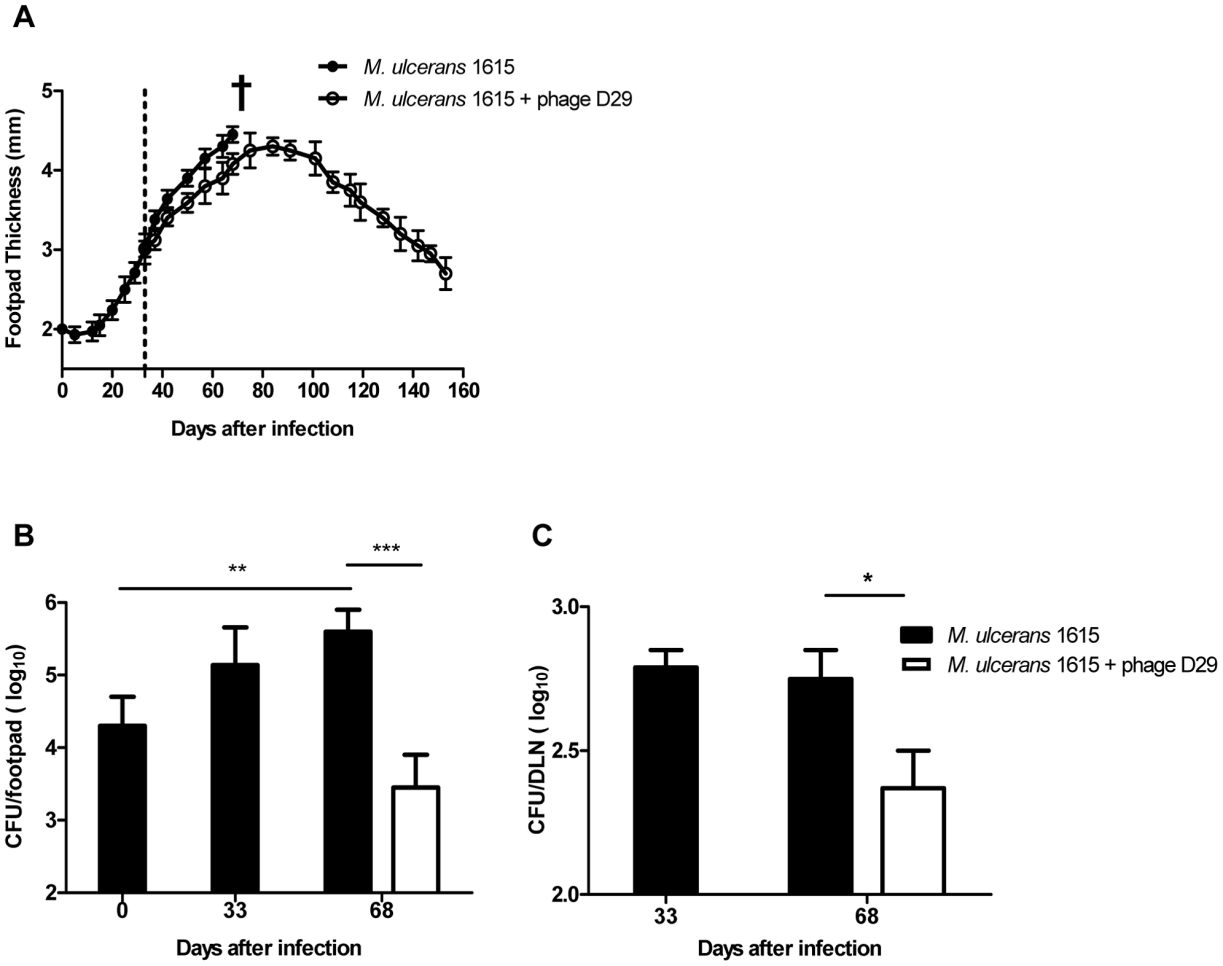
Lesion progression and *M. ulcerans* proliferation in the footpads and DLN of infected mice. Mice were infected subcutaneously in the left footpad with 5.5 log_10_ AFB of *M. ulcerans* strain 1615. After the emergence of macroscopic lesion (33 days post infection; footpad swelling of 3.0 mm) mice were subjected to treatment with a single dose of subcutaneous injection of mycobacteriophage D29 (dashed line). Lesion progression was assessed by measurement of footpad swelling (panel A) (n = 15). Bacterial proliferation was assessed by colony forming units in footpads (panel B) and in DLN (panel C) (n = 5). †, mice were sacrificed for ethical reasons after the emergence of ulceration of non-treated mice (68 days post infection). Results are from one representative experiment of two independent experiments. Data points and bars represent the mean ± SD (n = 5). Significant differences between treated and non-treated mice were performed using Student's t test (*, p≤0.05, **, p≤0.01, ***, p≤0.001).

To investigate the efficacy of mycobacteriophage D29 treatment for the control of *M. ulcerans*, we used a footpad mouse model of infection [34], [38], [39]. Mice were subcutaneously infected in footpads with 5.5 log_10_ AFB of *M. ulcerans* strain 1615. At day 33 post-infection, when footpad swelling had reached 3.0 mm (Figure 1A), mice were subcutaneously injected in the footpad with a single dose of mycobacteriophage D29 (8 log_10_ PFU) or with the vehicle MPB as a control.

In both control-infected and mycobacteriophage D29 treated mice we observed an initial footpad swelling (Figure 1A). However, at day 68 post-infection, footpads of non-treated mice started showing signs of ulceration, while in mycobacteriophage D29 treated mice the progression of swelling halted after day 91 post-infection (day 58 post-treatment) (Figure 1A). Furthermore, in mycobacteriophage D29 treated mice, we observed a progressive reduction of footpad swelling, until initial treatment values, recorded by day 150 post-infection. Moreover, signs of ulceration were continuously absent during the period of experimental infection (Figure 1A). The administration of mycobacteriophage D29 or vehicle MPB alone did not induce significant swelling of the footpad (data not shown).

Regarding *M. ulcerans* growth in infected footpads of non-treated mice, we observed a significant bacterial proliferation over the course of experimental infection (P<0.01) (Figure 1B). On the other hand, in footpads of mycobacteriophage D29 treated mice, we observed a significant reduction in CFU counts (P<0.001) at day 68 post-infection (day 35 post-treatment), following the administration of a single dose of mycobacteriophage D29 on day 33 post-infection (Figure 1B).

As previously described [38]–[40], we found that *M. ulcerans* disseminates to the DLN after footpad infection (Figure 1C ), probably due to continuous lymphatic dissemination of bacteria either freely or shuttled within phagocytes. Here we show a significant reduction in CFU counts (P<0.05) in the DLN of mycobacteriophage D29 treated mice, as compared with non-treated counterparts, at day 68 post-infection (day 35 post-treatment) (Figure 1C), correlating with the reduction of *M. ulcerans* numbers in the footpads.

### Mycobacteriophage D29 disseminates from footpad to the DLN

It is well known that bacteriophages can disseminate from the administration site and reach several organs such as lymph nodes, spleen and liver, which are the primary sites involved in phage clearance [41], [42]. In order to investigate the possible dissemination of mycobacteriophage D29, we determined phage titres in the footpad, DLN, spleen and blood after its inoculation in *M. ulcerans* infected footpads.

**Figure 2:**
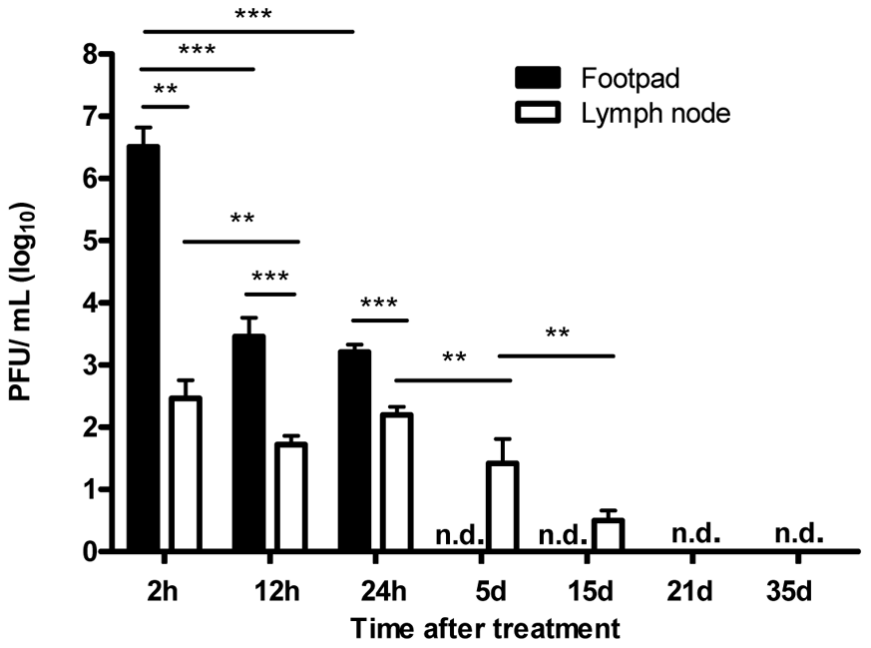
Mycobacteriophage D29 dissemination in footpads and DLN of mycobacteriophage D29-treated mice. Mice were infected subcutaneously in the left footpad with 5.5 log_10_ AFB of *M. ulcerans* strain 1615. After the emergence of macroscopic lesion (33 days post infection; footpad swelling of 3.0 mm) mice were subjected to treatment with a single dose of subcutaneous injection of mycobacteriophage D29. Phage titres were assessed by plaque forming units. n.d., not detected. Results are from one representative experiment of two independent experiments. The bars represent the mean ± SD (n = 5). Significant differences were performed using Student's t test (**, p≤0.01, ***, p≤0.001).

As shown in Figure 2, mycobacteriophage D29 numbers significantly decreased (P<0.001) in infected footpads from 2 to 24 h post-treatment and no phages could be detected after this time point (Figure 2). Phage numbers were also detected in the DLN, as early as 2 h after the administration in infected footpads, time point at which maximum phage counts were obtained (Figure 2). After 24 h, we observed a significant decrease (P<0.001) in phage titers in the DLN, but phages were still present until day 15 post-treatment (Figure 2). No phages could be detected in the DLN by the end of the experimental period of infection (day 35 post-treatment) (Figure 2).

D29 phages were also detected in the spleen (2.2 log_10_±0.25) and in the serum (2.3 log_10_±0.17) of mycobacteriophage D29 treated mice as early as 2 h post-treatment but were no longer detectable until the end of the experimental period.

### Increased levels of TNF, IFN-γ, and IL-10, but not IL-6, were induced in the footpad and DLN following treatment with mycobacteriophage D29

To characterize the profile of the immune response in *M. ulcerans*-infected tissues and to determine how phage treatment influences the host response, we carried out a comparative analysis of cytokine kinetics in DLN and footpads.

**Figure 3:**
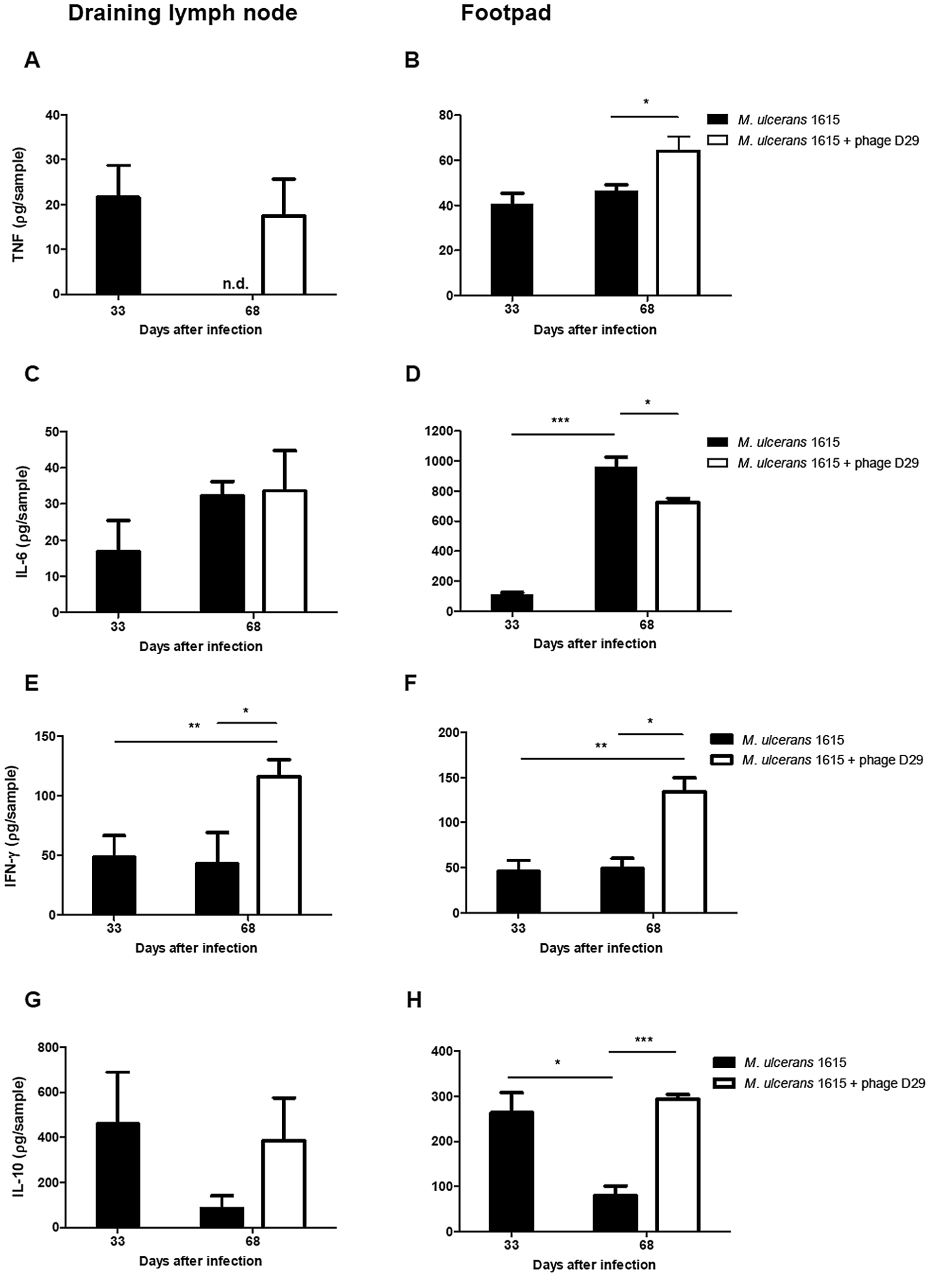
Cytokine profile in DLN and footpads of non-treated mice or mycobacteriophage D29-treated mice. Mice were infected subcutaneously in the left footpad with 5.5 log_10_ AFB of *M. ulcerans* strain 1615. After the emergence of macroscopic lesion (33 days post infection; footpad swelling of 3.0 mm) mice were subjected to treatment with a single dose of subcutaneous injection of mycobacteriophage D29. Levels of the TNF (panel A and B), IL- 6 (panel C and D), IFN-γ (panel E and F) and IL-10 (panel G and H) in DLN (panel A, C, E and G) and footpads (panel B, D, F and H) of mice were quantified by ELISA assay. n.d., not detected. Results are from one representative experiment of two independent experiments. Bars represent the mean ± SD (n = 5). Significant differences between treated and non-treated mice were performed using Student's t test (*, p≤0.05, **, p≤0.01, ***, p≤0.001).

Regarding the production of the pro-inflammatory cytokine tumor necrosis factor (TNF) in the DLN, at the emergence of ulceration, protein levels were no longer detectable in non-treated mice. In comparison, in mycobacteriophage D29 treated mice, significant levels of TNF were detectable at day 68 post-infection (day 35 post-treatment) (Figure 3A). Treatment with mycobacteriophage D29 also resulted in a significant increase of TNF levels in footpads of *M. ulcerans* infected mice (P<0.01) at day 35 post-treatment (day 68 post-infection), as compared with non-treated mice (Figure 3B).

Protein levels of IL-6 were detected in DLN and footpads of *M. ulcerans* infected mice at day 33 post-infection (Figure 3C and D). At day 68 post-infection (35 days post-treatment), higher levels of IL-6 were detected in footpads of infected non-treated mice (P<0.01), as compared with mycobacteriophage D29 treated mice (P<0.05) (Figure 3D).

As shown in Figure 3E and F, treatment with mycobacteriophage D29 resulted in a significant increase in the levels of IFN-γ in both the DLN and footpads (P<0.05), at day 35 post-treatment (day 68 post-infection) as compared with non-treated mice (Figure 3E and F).

The production of the anti-inflammatory cytokine IL-10 was also increased in both DLN and footpads of mycobacteriophage D29 treated mice (Figure 3G and H), as compared to non-treated mice at day 68 post-infection.

### D29 treatment is associated with the development and maintenance of a local mononuclear inflammatory response to *M. ulcerans*

**Figure 4:**
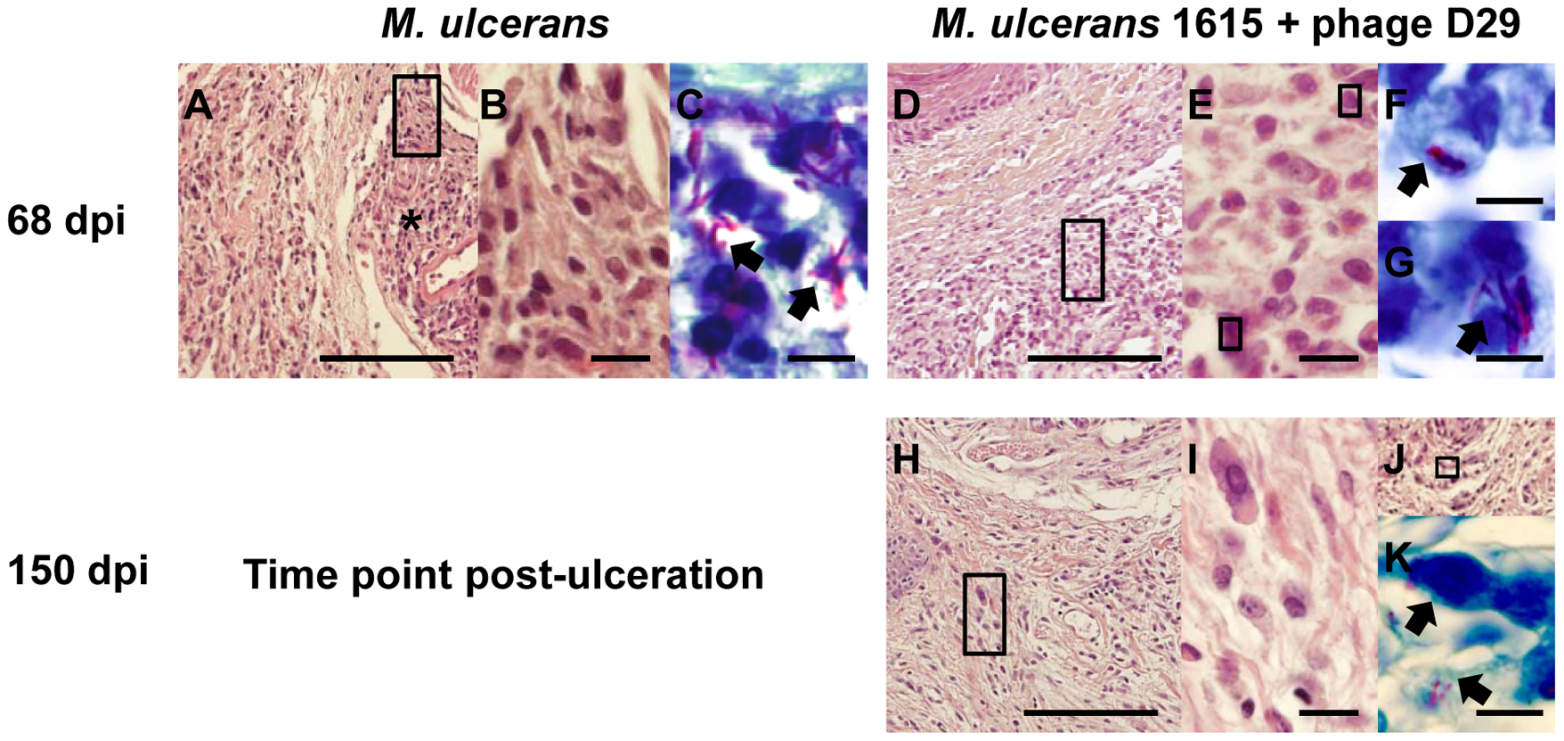
Histology of mice footpads of non-treated mice or mycobacteriophage D29-treated mice. Histological sections of footpads collected at different time points were stained with HE (A, B, D, E, H, I and J) or with ZN (C, F, G and K). For panels A, D, H and J, the scale bars represent 100 µm. For panels B, E and I, the scale bars represent 10 µm. For panels C, F, G and K the scale bars represent 5 µm. dpi, days post-infection. At 68 days post-infection (A–C), footpads of non-treated mice show necrotic areas (asterisks). Magnifications of panel A (rectangles) show mononuclear cells adjacent/in necrotic areas (B). Panel C show bacteria in necrotic areas (C; arrowheads). At day 35 after treatment (day 68 post-infection) (D–G), footpads of mycobacteriophage D29-treated mice show abundant cellular infiltration (D), composed mainly by mononuclear cells (E). Staining for bacteria in the same tissue areas and magnifications of the bacilli (arrowheads) are shown in panels F and G. At 150 days after treatment (H–K), footpads of mycobacteriophage D29-treated mice show a persistent inflammatory infiltrate (H–I). Staining for bacteria in remaining necrotic areas (J) are shown in panel K.

Histopathological analysis showed that at day 68 post-infection necrotic lesions (Figure 4A) were well established in the footpad tissue, as previously described in *M. ulcerans* progressing lesions from both humans and mice [34], [43]. Necrotic tissue was surrounded by an inflammatory infiltrate composed mainly by macrophages (Figure 4B). These necrotic areas, as expected, contained clumps of extracellular bacilli correlating with the emergence of footpad ulceration (Figure 4C). At the same time point (day 35 after treatment) in mycobacteriophage D29 treated mice, we observed an abundant cellular infiltration (Figure 4D) with a predominance of lymphocytes and macrophages (Figure 4E). We also observed bacilli, but they mainly co-localized with cells (Figure 4F and G). In addition, the maintenance of these inflammatory infiltrates (Figure 4H) mainly composed by mononuclear cells (Figure 4I), was observed 5 months after the end of mycobacteriophage D29 treatment. Although some bacilli were observed in the remaining necrotic areas (Figure 4J), as well at the periphery (Figure 4K), they were poorly stained by ZN.

To determine the effect of D29 phage inoculation, a group of mice was injected only with the phage. The histological analysis shows no significant alterations in subcutaneous tissues of non-infected mice inoculated with mycobacteriophage D29, at least until the end of the experimental period (day 150 after treatment) (data not shown).

### D29 treatment prevents DLN destruction

**Figure 5:**
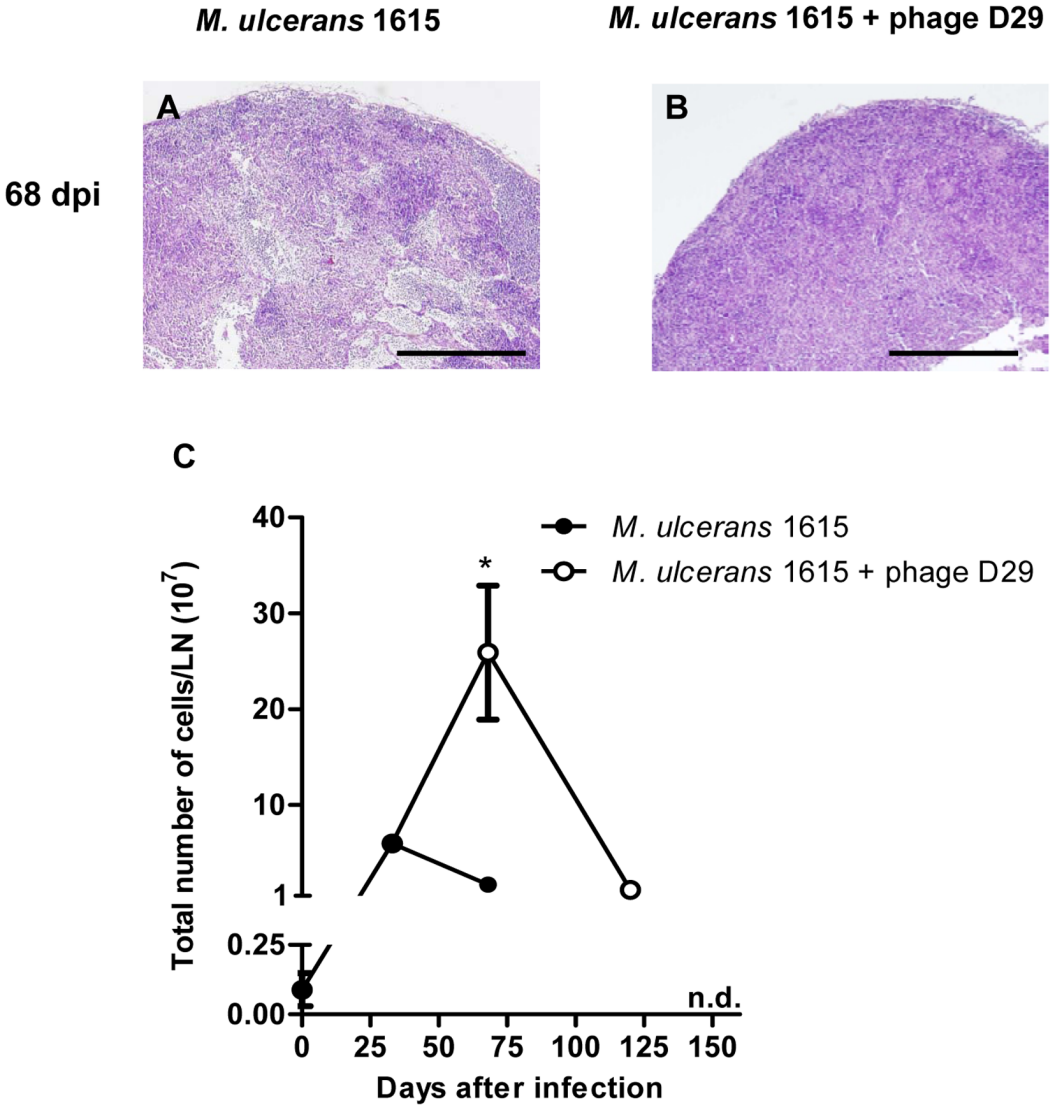
Histology and leukocyte kinetics in DLN of non-treated mice or mycobacteriophage D29-treated mice. Histological sections of DLN collected at different time points were stained with HE. For panels A and B the scale bars represent 500 µm. At 68 days post-infection DLN of non-treated mice show severe damage of the lymphoid tissue (panel A). At day 35 after treatment (day 68 post-infection), DLN structure of mycobacteriophage D29 treated animals was maintained (panel B). Total number of cells in the DLN was determined in DLN suspensions (panel C). Results are from one representative experiment of two independent experiments. n.d., not determined. In panel C data points represent the mean ± SD (n = 5). Significant differences between treated and non-treated mice were performed using Student's t test (*, p≤0.05).

Analysis of histopathology at day 68 post-infection showed that, in non-treated animals, the structure of the DLN was damaged, with absence of organized germinal centers leading to the destruction of the lymphoid tissue (Figure 5A), as recently reported in experimental *M. ulcerans* infection [38]. On the other hand, in D29 phage-treated mice the structure of the DLN was maintained with mild alterations (Figure 5B).

Previous studies from our laboratory showed that the initial increase of cell numbers in the DLN, upon footpad infection by *M. ulcerans*, is followed by a rapid decrease, correlating with the destruction of lymphoid tissue [38], [39].

Confirming previous results, here we observed a significant peak in the total cells (P<0.05) at day 33 post-infection, followed by a sharp decrease observed at day 68 post-infection (Figure 5C). We now show that mycobacteriophage D29 treatment induced a significant increase in the total number of cells in the DLN (P<0.05) at day 68 post-infection (day 35 after treatment).

## Discussion

The RS regimen for BU, recommended by the WHO [11], is effective for small lesions but presents several limitations and adverse side effects. Additionally, the RS regimen presents a variation in efficacy for advanced ulcerative stages of the disease, for which the adjunction of surgical resection of the infected skin followed by skin graft is often required [44].

The use of bacteriophages in targeting bacteria, even antibiotic resistant ones, has been regarded as an alternative method to control bacterial infections in both animals and humans [20]–[31], [45].

In fact, some studies have applied phage therapy to prevent and treat bacterial human diseases, such as the use of a novel, biodegradable preparation capable of releasing bacteriophages and ciprofloxacin (PhagoBioderm™), successfully used for the treatment of patients with severe radiation burns infected with multidrug-resistant *Staphylococcus aureus* [28]. In addition, early studies suggest that phage therapy may have potential for the treatment of mycobacterial diseases. Indeed, a reduction of lesions in the spleen, lungs and livers has been reported in experimentally infected guinea pigs with disseminated tuberculosis following therapy with phage DS-6A [46].

Previous reports suggest the potential use of mycobacteriophage D29 for the detection of *M. ulcerans* or for the assessment of drug resistance among mycobacterial isolates [36], [46]. In this study, we have demonstrated for the first time the potential of phage therapy against *M. ulcerans* infection. Indeed, we have shown in the mouse footpad model that a single subcutaneous injection of the lytic mycobacteriophage D29 can effectively decrease the proliferation of the mycolactone-producing *M. ulcerans* 1615. Importantly, mycobacteriophage D29 also showed lytic activity against several other *M. ulcerans* isolates *in vitro*, indicating that its activity *in vivo* may not be limited to *M. ulcerans* 1615.

As described, intravenous injection of phages enables a fast and directed introduction of phages in blood circulation and their spread through the organism [42]. Additionally, it has been described in mice that phages can also reach several organs, including lungs, kidney, spleen, liver and brain within 24 h after administration by other routes, including oral and traqueal routes [42]. Based on these observations, we studied the dissemination of mycobacteriophage D29 after subcutaneous injection in infected footpads. We show that mycobacteriophage D29 could only be detected in the blood and spleen of mice at 2 h post-injection, while in the footpad phages were detected until 24 h after injection. On the other hand, phages could be found in the DLN for longer periods of time, remaining viable for at least 15 days. The rapid elimination of phages from the circulation and their retention in the DLN as observed in our study, may be responsible for reducing the number of phages to a level that prevents complete bacterial clearance in infected footpads.

One possible approach to solve this rapid phage clearance, observed in both the footpads and the blood, may be through the administration of a long-lived circulating phage strain, as described in the case of other infection models [22], [47], [48].

Although using a high phage dose could also result in a decrease of phage clearance, studies have shown that this approach may result in bacterial death without phage replication [47], [49], [50] and also lead to a drop in the phage titer, effectively diminishing the dose of active phages.

Additionally, phage replication only occurs when the bacterial density is above a certain threshold [51]. This threshold is reached in the course of systemic infections [22], [48], [52], but may be compromised in the case of necrotic lesions, such as those induced by *M. ulcerans* infections. As described in phage treatment of a local *S. aureus* infection, even with multiple subcutaneous doses of 10^9^ PFU/mouse, phages significantly reduced but did not eliminate the bacterial load in abscesses induced by bacteria [22].

A possible concern about phage therapy is the emergence of phage-resistant bacteria [22], [24], [48], [53]. Although in this study we do not provide data related to the emergence of *M. ulcerans* phage-resistance, it has been described, in experimental models of other bacterial diseases, namely with *Pseudomonas aeruginos*a, *Escherichia coli* and *S. aureus*, that phage resistance is a rare event [22], [24], [48], even more so than antibiotic resistance [22], [54]. Even though we cannot rule out that some phage resistance can occur, the use, in this study, of a single phage treatment dose greatly reduces this hypothesis.

To characterize the type of immune response associated with the administration of mycobacteriophage D29 and, particularly, how phage treatment influences the host immune response against *M. ulcerans*, we carried out a comparative analysis of cytokine kinetics in footpads and DLN, where the initiation of the adaptive immune response occurs [38]. It is known that the differentiation/proliferation of mycobacteria-specific lymphocytes can occur in the DLN, early after *M. ulcerans* infection, and that effector T cells are recruited to the site of infection [38], where they mediate partial protection by enhancing IFN-γ-induced macrophage antimicrobial mechanisms. In agreement, we detected IFN-γ in the DLN, however this host response is not sufficient to inhibit the proliferation of virulent *M. ulcerans*, as increasing concentrations of mycolactone impair the effector activity of macrophages [6]. Interestingly, we observed that mycobacteriophage D29 treatment results in a significant increase in the total number of cells in the DLN, as well as in an increase of IFN-γ levels, correlating with a decrease in the number of viable bacteria, both in footpads and DLN, measured at day 68 post-infection (day 35 post-treatment). Collectively, these results suggest that the dissemination and prolonged permanence of phages in the DLN may prevent local *M. ulcerans* proliferation and the associated accumulation of mycolactone, therefore preventing DLN destruction.

As previously described [38]–[40] and confirmed in this study, the tissue destruction of the DLN was associated with bacterial colonization, which is consistent with the spreading of *M. ulcerans* from the site of infection via afferent lymphatic drainage [55], [56]

On the other hand, the increased immune activation induced in the DLN of treated mice may explain an immune-mediated control of bacterial proliferation in the footpad, despite the lack of phages at the primary site of infection. In fact, as previously described, IFN-γ, and TNF play a protective role in immunity against *M. ulcerans* experimental infections, contributing to control bacterial proliferation [6], [7], [57]. Accordingly, we show here that mycobacteriophage D29 treatment was associated with increased levels of both IFN-γ and TNF in *M. ulcerans*-infected footpads [6], [7], correlating with a predominance of a mononuclear infiltrate and prevention of ulceration at 150 days post-infection. Additionally, our histological data show that bacilli are still present in footpad tissues, albeit with an altered morphology and poorly stained with ZN. Although this observation may indicate that, as described [39], *M. ulcerans* bacilli underwent degradation after bacterial killing, a possible relapse of *M. ulcerans* infection after the 150 day period of experimental infection was not checked in this study.

Here we show that IL-6 concentration was markedly lower in footpads of mycobacteriophage D29 treated mice at day 68 post-infection (day 35 after treatment) as compared to non-treated mice, confirming that footpad tissue damage is less severe in D29 phage treated footpads.

Although production of IL-10 could be detected in skin lesions of patients with BU [58], [59] the exact role of this cytokine in the progression of *M. ulcerans* infection has to be further analyzed. In this experimental setting, it is possible that the increased levels of IL-10 in mycobacteriophage D29 treated mice are modulating the activity of the pro-inflammatory cytokines.

In summary, our results show that administration of the lytic mycobacteriophage D29: (i) is an effective approach for reducing *M. ulcerans*-induced pathology in the mouse model of infection; (ii) reduced *M. ulcerans* numbers in the footpad and the DLN, associated with increased IFN-γ and TNF levels and; (iii) is not associated with detectable side effects over a minimum delay of 150 day observation period.

To our knowledge, this is the first study on mycobacteriophage therapy against *M. ulcerans in vivo* infection. It should be pointed out that mice were treated at an advanced stage of *M. ulcerans* infection, which is relevant for human infection since BU patients often seek medical treatment in advanced stages of the disease. More detailed studies examining the effects of phage dosage, routes and timing of administration, as well as on pharmacokinetics, will be needed to determine if phage therapy will provide a consistent alternative/supplement for the treatment of BU. Although the development of a therapeutic regimen using phages will involve a commitment to fulfill the scientific requirements of current pharmaceutical agencies, our encouraging results justifies further investigation on the potential of phages for the management of this mycobacteriosis. Moreover, mycobacteriophage D29 represents an ideal agent from a regulatory standpoint in that it has been fully characterized genetically [60] and is able to be used on a stand-alone basis. Another approach could be based on the therapeutic use of lysins bacteriophage proteins produced at the end of a lytic life cycle, designed to attack peptidoglycan in order to allow the release of the new synthesized phage particles [61].
